# Bumble bees exhibit body size clines across an urban gradient despite low genetic differentiation

**DOI:** 10.1038/s41598-022-08093-4

**Published:** 2022-03-09

**Authors:** Matthew W. Austin, Amber D. Tripodi, James P. Strange, Aimee S. Dunlap

**Affiliations:** 1 Living Earth Collaborative, Washington University in St. Louis, 320 McDonnell Hall, Danforth Campus, St. Louis, MO 63105 USA; 2 Department of Biology, University of Missouri-St. Louis, St. Louis, MO USA; 3 Whitney R. Harris World Ecology Center, St. Louis, MO USA; 4 USDA-ARS-Pollinating Insect Research Unit, Logan, UT USA; 5 Department of Entomology, The Ohio State University, Columbus, OH USA

**Keywords:** Ecology, Molecular ecology, Urban ecology

## Abstract

Environmental heterogeneity resulting from human-modified landscapes can increase intraspecific trait variation. However, less known is whether such phenotypic variation is driven by plastic or adaptive responses to local environments. Here, we study five bumble bee (Apidae: *Bombus*) species across an urban gradient in the greater Saint Louis, Missouri region in the North American Midwest and ask: (1) Can urban environments induce intraspecific spatial structuring of body size, an ecologically consequential functional trait? And, if so, (2) is this body size structure the result of plasticity or adaptation? We additionally estimate genetic diversity, inbreeding, and colony density of these species—three factors that affect extinction risk. Using ≥ 10 polymorphic microsatellite loci per species and measurements of body size, we find that two of these species (*Bombus impatiens*, *Bombus pensylvanicus*) exhibit body size clines across the urban gradient, despite a lack of population genetic structure. We also reaffirm reports of low genetic diversity in *B. pensylvanicus* and find evidence that *Bombus griseocollis*, a species thought to be thriving in North America, is inbred in the greater Saint Louis region. Collectively, our results have implications for conservation in urban environments and suggest that plasticity can cause phenotypic clines across human-modified landscapes.

## Introduction

In the Anthropocene, we have witnessed precipitous declines of biodiversity^1^, with approximately 1 million species currently in threat of extinction^2^. Anthropogenic effects on the globe are widely recognized as the primary drivers of this biodiversity loss^2^. Humans have transformed up to one-half of global land surfaces^3^, thereby fragmenting previously continuous habitat and presenting many species with environments unencountered in their evolutionary past^4^. Anthropogenic change may increase extinction risk by inducing mismatch between functional traits and the environment, if such traits are not sufficiently plastic^5^. Additionally, by creating barriers to dispersal, such habitat fragmentation may induce genetic differentiation among isolated subpopulations and loss of genetic diversity within them, which can further exacerbate population declines^6^. As functional traits mediate population performance via effects on fitness^7^, while population genetics indicate long-term population stability^8^, effective conservation efforts are strengthened by integrative assessments of population genetics and how functional traits are distributed in human-modified environments.

Biodiversity loss among pollinating insects is particularly important for empirical inquiry, as insects are primarily responsible for the pollination of wild plants and agricultural crops^9^. Of the pollinating insects, the most comprehensive estimates of decline are for bees (Anthophila)^10^ and butterflies (Rhopalocera)^11^. Various bee taxa have experienced range contractions^12^, abundance declines^12^, and local extinctions^13,14^, thereby resulting in species richness losses. Among these taxa are the bumble bees (Hymenoptera: Apidae: *Bombus*), a monophyletic group of eusocial bees primarily native to temperate and subpolar regions of the Northern Hemisphere^15^. Bumble bees have undergone precipitous declines throughout their native range^16,17^, with estimates suggesting that approximately one-third of bumble bee species are in decline^18^. Anthropogenic habitat modification is widely recognized as a predominant driver of these declines^10^, with habitat loss reducing the availability of forage and nesting sites^10^, fragmentation inducing heterogeneity in species occurrences^19^, and population success differing between rural and urban areas^20^.

Previous studies have demonstrated that human-modified environments can structure bee communities interspecifically, based on the matching of functional traits to local environments^21,22^. However, less known is whether human-modified environments can structure bee functional traits intraspecifically. The literature is increasingly documenting that species may respond to human-induced environmental heterogeneity by increasing intraspecific trait variation^23^. In bees, body size is one functional trait that has considerable ecological consequences. At the community-level, body size influences pollination system connectivity by dictating the floral species from which a bee can forage^24^. This is predominantly due to allometric scaling between bee body size and tongue length, and the functional match between tongue length and corolla tube length^24^. At the individual-level, body size influences a suite of characteristics, including dispersal distance^25^, foraging efficiency^26^, and resistance to starvation^27^. In bumble bees, body size is developmentally plastic, with higher rates of larval feeding yielding larger adult workers^28,29^. This plasticity can result in up to tenfold differences in worker body size within colonies, despite workers from monogamous queens being highly related (*r* = 0.75)^15,30^. Furthermore, body size may influence bumble bees’ susceptibility to decline; species with larger average body size^31^ or lower variation in body size^32^ appear more susceptible to negative effects of human activity. Despite the known ecological implications of bumble bee body size, we lack a comprehensive understanding of how body size can be structured within-species across human-modified environments.

Conservation efforts are strengthened by considering how functional traits are distributed across heterogeneous landscapes^23^. Understanding the link between environment and phenotype is critical for habitat restoration^33^ and species relocations^34^. Additionally, phenotypic divergence between subpopulations may indicate variance in environmental quality and differential extinction risk among subpopulations^35^. Coupling functional trait investigations with population genetics can elucidate whether phenotypic divergence mirrors patterns of population genetic structuring^36^. If these mirror one another, phenotypic divergence may indicate divergent selection between subpopulations, while phenotypic divergence without genetic structure may indicate plasticity in local environments despite high rates of gene flow^36^. This is important as divergent selection can alter the delineation of evolutionarily significant units^37^ and the degree to which functional traits are plastic can affect range shifts, extinction, and persistence of threatened species^5,38^. Conservation efforts can be further strengthened by population genetics by estimating factors that may contribute to extinction risk, including inbreeding, reduced genetic diversity, and low effective population size^39^. Various conservation-genetic techniques have been developed to study bee ecology and evolution^40^. Genotyping of microsatellites has proven particularly versatile^6,41^ and is a robust method for detecting genetic effects of recent habitat fragmentation, even in species with high gene flow^42^.

Here, we investigate body size spatial structuring and population genetics in five bumble bee species across the greater Saint Louis, Missouri region: *Bombus auricomus*, *Bombus bimaculatus*, *Bombus griseocollis*, *Bombus impatiens*, and *Bombus pensylvanicus*. These species have experienced divergent population trends over the past two centuries in North America; *B. auricomus* and *B. pensylvanicus* have decreased relative abundance, while *B. impatiens*, *B. bimaculatus*, and *B. griseocollis* have experienced abundance increases^17^. The International Union for Conservation of Nature (IUCN) Red List categorizes all of these species as “Least Concern” with stable population trends, except for *B. pensylvanicus*, which is listed as “Vulnerable” with a declining population trend^43^. Recent data suggest a listing of “Critically Endangered” for *B. pensylvanicus* in Canada, following IUCN Red List criteria^44^. In addition to body size being intraspecifically variable, these species also exhibit marked interspecific differences in worker body size^32^. Bumble bees are typically regarded as dietary generalists^15^; however, given that body size influences the floral species that bees can forage from^24^, these interspecific size differences may result in a degree of resource partitioning between species. By estimating population genetics using microsatellites and analyzing intraspecific spatial structure of body size, we provide an integrative, comparative assessment of conservation genetics and trait variation in a group of at-risk pollinating insects. We ask the following questions: (1) do these species exhibit intraspecific spatial structure in body size across an urban gradient and, if so, (2) is this body size structure the result of plastic or adaptive responses to local environments? We additionally estimate genetic diversity, inbreeding, and colony density for these species throughout the greater Saint Louis region, as these factors can help inform conservation efforts. As anthropogenic changes to the biosphere continue to drive biodiversity loss, it is of paramount importance to understand functional trait variability and conservation genetics of groups at risk of extinction.

## Methods

### Study sites and sampling

**Figure 1:**
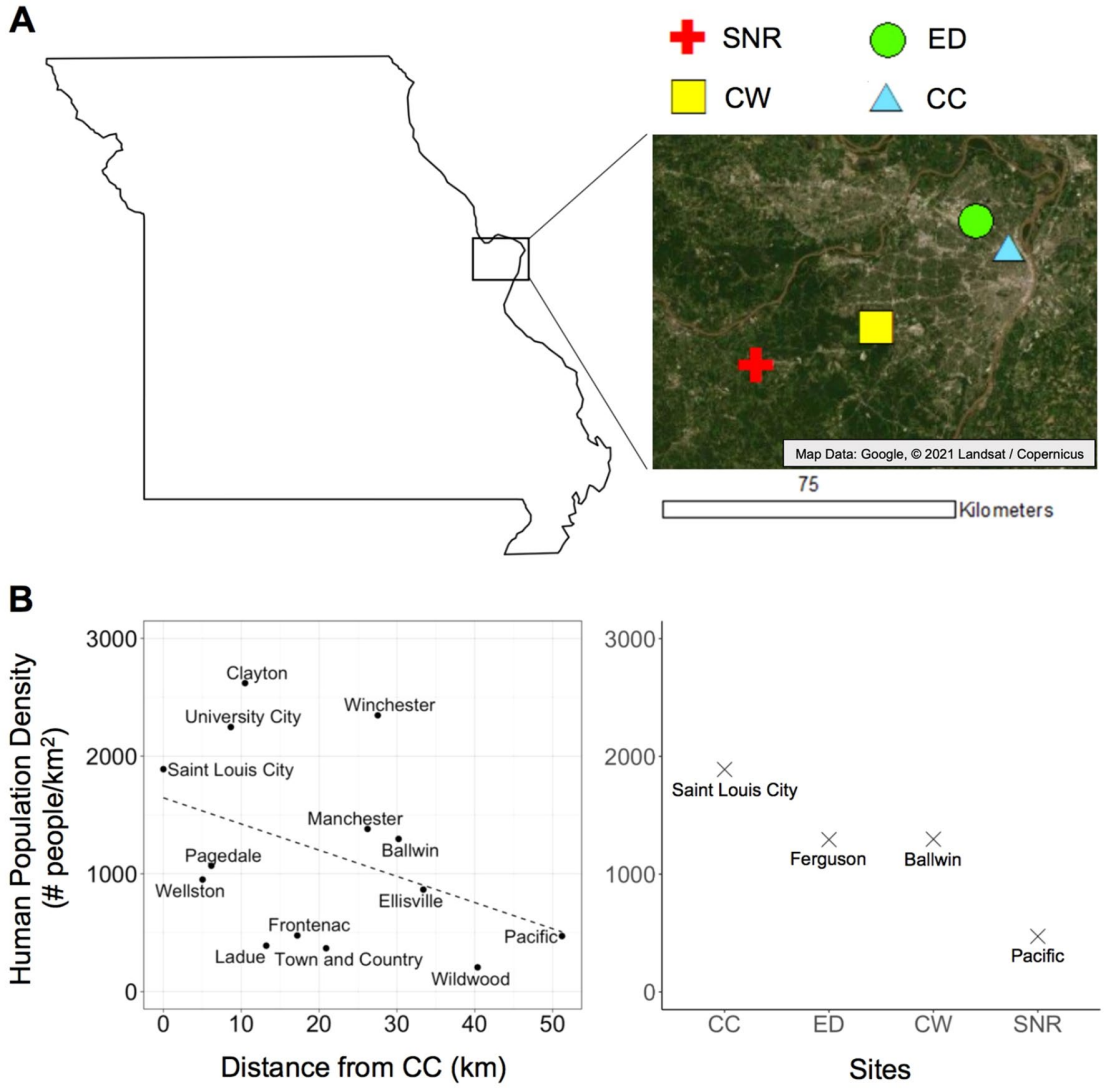
(**A**) Map of sampling locations. *CC* Calvary Cemetery, *CW* Castlewood State Park, *ED* EarthDance Farms, *SNR* Shaw Nature Reserve. Map generated with Google Earth 9.154.0.1 (https://earth.google.com). (**B**) Human population density per locality. Left panel: Urban gradient depicted by human population density per locality from CC (Saint Louis City, MO) to SNR (Pacific, MO). Distance from CC is the distance from CC to the approximate midpoint of a locality that occurs along the trajectory from CC to SNR. Right panel: Human population density of each locality where a site is located.

We sampled bumble bees in the greater Saint Louis, Missouri region in 2018, throughout the entire period of colony activity for each species. The five focal bumble bee species in this study (*B. auricomus*, *B. bimaculatus*, *B. griseocollis*, *B. impatiens*, *B. pensylvanicus*) can all be reliably found throughout this area^45^. We sampled bumble bees weekly from each of four sites: Calvary Cemetery (CC), EarthDance Farms (ED), Castlewood State Park (CW) (permission by Missouri Department of Natural Resources, Application for Research in Missouri State Parks 2018; Christopher Crabtree *personal communication*), and Shaw Nature Reserve (SNR) (Fig. 1A). These sites occur along a gradient from Saint Louis city to an area west of Saint Louis, which follows a trend of decreasing human population density (number of people km^−2^) with increased distance from Saint Louis (Fig. 1B). To calculate human population density, we used data on cities and towns from the United States Census Bureau^46,47^. We used population estimates for July 1st, 2018^47^ as measures of human population size per locality and land area (converted to km^2^^46^) as measures of total area per locality that a human population may occupy. We calculated human population density as the average number of people km^−2^, by dividing population estimates by land area. Human population density is a commonly used metric for anthropogenic influence on the environment^48,49^; therefore, we consider our sites as occurring along an urban gradient, where sites occurring in localities with greater human population density are considered more urban (Fig. 1B; see Supplemental Materials for site descriptions). As the minimum distance separating any two of these sites is greater than the typical dispersal distance of queen bumble bees^50^, we treat all conspecific bees per individual site as a putative subpopulation.

We opportunistically collected bees by hand-netting and immediately transferred them to individual ventilated vials. For all bees collected while actively foraging on a flower, we recorded the floral genus the bee was foraging on. We employed non-lethal sampling^51^ and released bees following data collection. Before release, we identified bees to species and sex following color patterning and morphological descriptions of Williams et al.^52^, removed a mid-leg tarsus from each bee and immediately stored it below 0 °C in 100% ethanol for microsatellite genotyping. For a subset of bees, we also measured thorax width using digital calipers (standard practice for measurements of bee body size^15,53^) prior to release.

### Microsatellite genotyping

We performed DNA extraction and PCR amplification at the University of Missouri—St. Louis. Immediately prior to DNA extraction, we dried mid-leg tarsus samples and transferred each sample to a 96 well plate. In between samples, we immersed the forceps used for this work in 95% ethanol to prevent cross contamination. We followed a Chelex-based DNA extraction protocol^54^, whereby we added 150 μL Chelex 100 (Bio-Rad) and 5 μL Proteinase K (Apex Bioresearch) to each sample, and subsequently incubated samples in a Bio-Rad T100 Thermal Cycler with the following conditions: (1) 55 °C for 1 h, (2) 99 °C for 15 min, (3) 37 °C for 1 min, and (4) 99 °C for 15 min. Prior to PCR amplification, we stored extracted DNA samples at − 20 °C.

We genotyped each sample at 18 dye-labeled microsatellite loci^55–58^. Not all loci were successfully amplified or reliably scored within each species, so each species had its own complement of loci used for analyses (Table S1). We ran two multiplex PCRs per sample (i.e., plexes A and B), with six to nine microsatellite primers in each multiplex. Each multiplex reaction mixture contained 1 μL Chelex DNA extraction supernatant, 2 μL Promega 5 × buffer, 0.56 μL MgCl_2_ 25 mM (Promega), 0.6 μL dNTP (Promega), 0.2 μL bovine serum albumin (Promega), 0.08 μL Taq polymerase (Promega), 2.28–3.08 μL H_2_O, and 0.045–0.400 μL of each primer (forward labelled with VIC, NED, 6-FAM, or PET dyes; Applied Biosystems). Each sample had a total reaction mixture volume of 10 μL, contained in a new well of a 96 well plate. We performed each PCR using a Bio-Rad T100 Thermal Cycler with the following conditions: (1) 95 °C hot start, (2) initial denaturation at 95 °C for 3.5 min, (3) 31 cycles of 95 °C for 30 s, 55 °C (plex A) or 58 °C (plex B) for 1.25 min, 72 °C for 45 s, and (4) final extension of 72 °C for 15 min. Subsequently, we sent 2 μL of each PCR product to the University of Missouri DNA Core for fragment analysis, where DNA Core staff added formamide and an internal size standard (600 LIZ). We scored alleles using Geneious 11.0.4 with the Microsatellite Plugin^59^. Following microsatellite genotyping, we verified species identifications by confirming that each individual’s alleles fell within their species-specific allele bins. Furthermore, we discarded from downstream genetic analyses all individuals and loci with 20% or greater genotyping failure per species.

### Colony density

Measuring effective population size (*N*_e_) can be problematic in eusocial insects, as non-reproductive worker abundances can inflate *N*_e_, unless colony relationships are controlled for^60^. Therefore, we used colony density (*N*_c_) (i.e., effective colony number) as a measure of *N*_e_, which estimates the number of colonies at a site after controlling for colony relationships among workers^6,60,61^. We calculated *N*_c_ solely with female genotypes. Prior to estimating *N*_c_, we removed loci per species that had ≥ 25% null allele frequency following Chakraborty et al.^62^, using the R package PopGenReport 2.0^63^. We estimated *N*_c_ per subpopulation by first reconstructing female sibships in Colony 2.0^64^ using a 5% genotyping error rate and a 95% probability of females being full siblings. Following sibship reconstructions, we calculated *N*_c_ following Geib et al.^61^. To do so, we first determined the number of sampled females (*N*_i_), the number of successfully genotyped females (*N*_g_), and the number of colonies detected by Colony (*N*_nr_). We then calculated the number of colonies detected standardized for genotyping success as *N*_ns_ = (*N*_nr_/*N*_g_) × *N*_i_. Finally, we calculated *N*_c_ according to the Crozier model for effective population size of eusocial haplodiploid species that estimates detected colonies plus colonies not detected by sampling: *N*_c_ = (4.5*Nnm*)/(1 + 2*m*); *N* is detected colony number, *n* is queen number per colony, and *m* is mating frequency^65^. Accordingly, for species like bumble bees, that are characterized by monogyny and monoandry^15^, this calculation simplifies to *N*_c_ = 1.5 × *N*_ns_^6^. We did not calculate *N*_c_ for any subpopulation with 15 or fewer successfully genotyped females (i.e., *N*_g_ ≤ 15).

### Population genetic analyses

We included only one randomly chosen sister per colony for population genetic analyses. After retaining one sister per colony, we checked loci for linkage disequilibrium (LD) using the R package Genepop ‘007 1.1.4^66^. If we found two or more loci to be in significant LD (*p* < 0.05), we retained only one of these loci for further genetic analyses. We tested individual loci for Hardy–Weinberg equilibrium (HWE) using the R package PopGenReport 2.0^63^.

Following these quality control measures, we calculated allelic richness (i.e., mean allele number per locus; *AR*) per subpopulation and global *AR* per species (i.e., species-level *AR* grouping samples across sites). As *AR* can be sensitive to variances in sample size, sample size rarefaction is the preferred method of standardizing *AR* for comparative studies^67^. Prior to calculating *AR* values, we rarefied subpopulation sample sizes to the lowest subpopulation sample size across all five species, using the R package hierfstat 0.04-22^68^. For global measures of *AR*, we rarefied each species’ sample size to the sample size of the species with the lowest overall sample size.

To assess genetic differentiation among intraspecific subpopulations, we calculated *F*_ST_ across all loci per species^69^ in FSTAT 2.9.4. To ensure that our data had sufficient statistical power to detect true genetic differentiation, we performed a power simulation per species with the program POWSIM 4.1, which tests the null hypothesis of no genetic differentiation between subpopulations, given different combinations of sample size, loci, and alleles^70^. See Supplemental Materials for full power analysis methods.

We tested each species for possible inbreeding by (1) calculating the inbreeding coefficient, *F*_IS_, across all loci per species^69^ in FSTAT 2.9.4, and (2) inspecting males for diploidy. In bee populations, diploid male frequency increases with inbreeding due to increased rates of homozygosity at the complementary sex determination locus^71^. To assess male diploidy, for each male bee we recorded whether each successfully genotyped locus was scored as homozygous or heterozygous. Following Darvill et al.^72^, we then recorded a male as diploid if three or more of his loci were scored as heterozygous. For calculations of *F*_ST_, *F*_IS_, and subpopulation *AR*, we removed all individuals from populations with < 25 samples following our quality control measures^73^. However, we did not remove individuals from populations with a low sample size for our calculations of global *AR*, while still ensuring that only one randomly chosen sister per colony was included in these calculations.

### Body size variation analyses

For all body size variation analyses, we included only one randomly chosen sister per colony and excluded all subpopulations that included ≤ 15 workers with thorax width measurements. Given our weekly sampling protocol across sites, these measurements collectively represent body size variation across each species’ entire period of colony activity. To determine whether our focal bumble bee species exhibit intraspecific spatial structure in body size, we compared intraspecific subpopulations for significantly different average body sizes. We first ran an analysis of variance (ANOVA) with thorax width as the response variable, and site and species as categorical predictors. Subsequently, we ran contrasts between least squares means for each unique pairing of intraspecific subpopulations. We used a Bonferroni corrected *α*-value to determine statistical significance of these contrasts. To compute these contrasts, we used the R package lsmeans 2.30^74^.

## Results

### Sampling and genotyping

Across all species and sites, we collected 839 bees; 774 females and 65 males. Sample sizes were variable across species and sites (Tables 1 and 2), ranging from conspecific bees being absent or found in low abundance to upwards of 70 conspecific bees collected at a site. Following all genotyping quality control measures, each species had a minimum of 10 loci used in population genetic analyses (Fig. S1; Table S1). A description of these quality control results and loci retained per species can be found in the Supplemental Materials.

**Table 1:** Colony density estimates for bumble bee (*Bombus* spp.) subpopulations throughout the greater Saint Louis region in 2018. *N*_i_ is the total number of sampled females, *N*_g_ is the number of successfully genotyped females, *N*_nr_ is the number of colonies detected from genotyping, *N*_ns_ is the number of colonies standardized for genotyping success, and *N*_c_ is colony density. Colony numbers were not calculated for populations with 15 or fewer successfully genotyped females. *CC* Calvary Cemetery, *CW* Castlewood State Park, *ED* EarthDance Farms, *SNR* Shaw Nature Reserve.

Species and colony estimates	Sites			
	CC	CW	ED	SNR
***B. auricomus***				
*N*_i_	56	–	39	44
*N*_g_	54	–	38	38
*N*_nr_	54	–	36	36
*N*_ns_	56.0	–	36.9	41.7
*N*_c_	84.0	–	55.4	62.5
***B. bimaculatus***				
*N*_i_	1	72	49	38
*N*_g_	1	70	49	34
*N*_nr_	–	64	46	33
*N*_ns_	–	65.8	46.0	36.9
*N*_c_	–	98.7	69.0	55.3
***B. griseocollis***				
*N*_i_	45	12	61	34
*N*_g_	45	12	56	32
*N*_nr_	45	–	54	32
*N*_ns_	45.0	–	58.8	34.0
*N*_c_	67.5	–	88.2	51.0
***B. impatiens***				
*N*_i_	53	42	71	41
*N*_g_	48	42	64	39
*N*_nr_	45	41	58	35
*N*_ns_	49.7	41.0	64.3	36.8
*N*_c_	74.5	61.5	96.5	55.2
***B. pensylvanicus***				
*N*_i_	39	5	19	53
*N*_g_	38	5	16	52
*N*_nr_	28	–	11	44
*N*_ns_	28.7	–	13.1	44.8
*N*_c_	43.1	–	19.6	67.3

**Table 2:** Sample sizes and diploidy of male bumble bees (*Bombus* spp.) in the greater Saint Louis region in 2018. *N*_i_ is the total number of sampled males, *N*_g_ is the number of successfully genotyped males, *N*_d_ is the number of diploid males (i.e., number of males with ≥ 3 heterozygous loci). Percent diploid males is *N*_d_/*N*_g_. Each value is calculated per species by site and globally (i.e., combining all sites). *CC* Calvary Cemetery, *CW* Castlewood State Park, *ED* EarthDance Farms, *SNR* Shaw Nature Reserve.

Species and statistics	Sites				Global values
	CC	CW	ED	SNR	
***B. auricomus***					
*N*_i_	0	0	0	0	0
*N*_g_	–	–	–	–	–
*N*_d_	–	–	–	–	–
% Diploid	–	–	–	–	–
***B. bimaculatus***					
*N*_i_	0	10	8	5	23
*N*_g_	–	10	8	5	23
*N*_d_	–	0	0	0	0
% Diploid	–	0.00	0.00	0.00	0.00
***B. griseocollis***					
*N*_i_	1	9	13	2	25
*N*_g_	1	9	13	2	25
*N*_d_	1	9	10	1	21
% Diploid	100.00	100.00	76.92	50.00	84.00
***B. impatiens***					
*N*_i_	0	9	5	0	14
*N*_g_	–	8	3	–	11
*N*_d_	–	0	0	–	0
% Diploid	–	0.00	0.00	–	0.00
***B. pensylvanicus***					
*N*_i_	2	0	1	0	3
*N*_g_	2	–	1	–	3
*N*_d_	0	–	0	–	0
% Diploid	0.00	–	0.00	–	0.00

### Colony density

Each species had variable colony densities across sites. *N*_c_ ranges from a minimum of 19.6 (*B. pensylvanicus* at ED) to a maximum of 98.7 (*B. bimaculatus* at CW) (Table 1). We could not calculate *N*_c_ for *B. auricomus*, *B. griseocollis*, or *B. pensylvanicus* at CW, and for *B. bimaculatus* at CC, due to fewer than 15 females having been successfully genotyped for these subpopulations (i.e., *N*_g_ < 15) (Table 1).

### Population genetic analyses

Throughout the greater Saint Louis region, genetic differentiation between intraspecific subpopulations was low to absent in each species, with *F*_ST_ ≤ 0.002 in each species and all 95% CIs including zero (Table 3). Each power simulation revealed statistical power > 0.99 for detecting an *F*_ST_ = 0.05 using both Chi-square and Fisher’s exact tests. Accordingly, our sampling protocol had a > 99% probability of detecting true *F*_ST_ values of 0.05. *F*_IS_ values were more variable, ranging from a minimum of 0.023 (*B. bimaculatus*) to a maximum of 0.151 (*B. griseocollis*) (Table 3). Zero is only included in the *F*_IS_ 95% CI of *B. bimaculatus*. All males collected were haploid, except in *B. griseocollis* for which 21 of 25 collected males (84%) were diploid (i.e., ≥ 3 loci scored as heterozygous) (Table 2). Global *AR* calculations were rarefied to a sample size of 88 per species, following *B. pensylvanicus* having the lowest overall sample size (i.e., 88 female genotypes retained × 2 alleles/female = 176 alleles). Subpopulation *AR* calculations were rarefied to a subpopulation size of 28, as the subpopulation included in genetic analyses with the lowest sample size was *B. pensylvanicus* at CC (i.e., 28 female genotypes retained × 2 alleles/female = 56 alleles). *AR* varies interspecifically (i.e., between species’ global *AR* values) and between intraspecific loci (Table 3; Fig. S1). *Bombus pensylvanicus* had the lowest *AR* across all species (global *AR* = 6.29 ± 1.42 SE) and *B. impatiens* had the highest *AR* (global *AR* = 10.24 ± 2.21 SE). We could not calculate *F*_ST_, *F*_IS_, and site-specific *AR* for *B. auricomus*, *B. griseocollis*, or *B. pensylvanicus* at CW, *B. bimaculatus* at CC, and *B. pensylvanicus* at ED due to < 25 genotypes remaining in each of these subpopulations following our quality control measures.

**Table 3:** Population genetic statistics for bumble bees (*Bombus* spp.) in the greater Saint Louis region. Allelic richness (*AR*), calculated as mean allele number across loci, is calculated per species for each site and combining all sites (i.e., global *AR*). *F*_ST_ describes population genetic differentiation. *F*_IS_ is the inbreeding coefficient. All populations with < 25 successfully genotyped individuals following quality control measures were removed from population genetic analyses. *SE* standard error, *95% CI* 95% confidence interval, *CC* Calvary Cemetery, *CW* Castlewood State Park, *ED* EarthDance Farms, *SNR* Shaw Nature Reserve.

Species	*AR* (SE) per Site				Global *AR* (SE)	*F*_ST_ (95% CI)	*F*_IS_ (95% CI)
	CC	CW	ED	SNR			
*B. auricomus*	7.24 (1.54)	–	7.05 (1.53)	7.41 (1.67)	8.94 (1.92)	0.002 (− 0.004 to 0.010)	0.075 (0.031 to 0.124)
*B. bimaculatus*	–	7.25 (1.21)	7.12 (1.32)	7.24 (1.39)	9.25 (1.63)	0.000 (− 0.002 to 0.002)	0.023 (− 0.028 to 0.072)
*B. griseocollis*	6.76 (1.57)	–	6.43 (1.58)	6.52 (1.65)	8.61 (2.05)	0.002 (− 0.002 to 0.008)	0.151 (0.090 to 0.203)
*B. impatiens*	7.77 (1.80)	8.20 (1.98)	7.76 (1.85)	8.15 (1.69)	10.24 (2.21)	0.001 (− 0.001 to 0.004)	0.068 (0.025 to 0.122)
*B. pensylvanicus*	5.57 (1.32)	–	–	4.92 (1.13)	6.29 (1.42)	− 0.003 (− 0.008 to 0.002)	0.070 (0.008 to 0.140)

### Body size variation analyses

**Figure 2:**
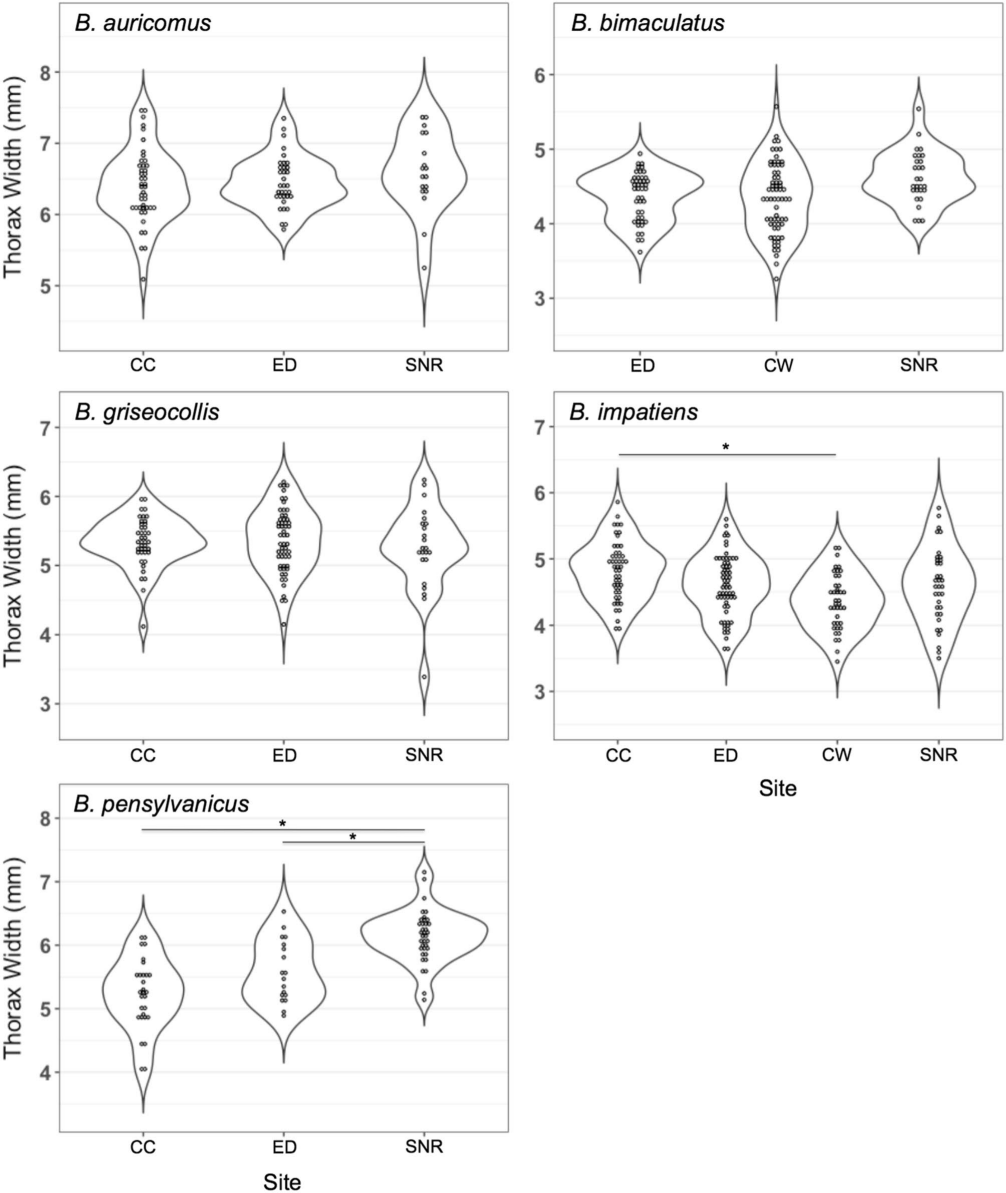
Thorax widths of worker bumble bees (*Bombus* spp.) across the urban gradient in the greater Saint Louis region. Sites are arranged from left to right by increasing distance from Saint Louis City. Asterisks (*) indicate statistically significant differences between means of intraspecific subpopulations following Bonferroni correction (i.e., *p* < 0.00278). *CC* Calvary Cemetery, *CW* Castlewood State Park, *ED* EarthDance Farms, *SNR* Shaw Nature Reserve.

We find evidence for spatial structuring of intraspecific body size for bumble bees in the greater Saint Louis region. Our full ANOVA revealed significant effects of species, site, and their interaction on worker thorax width (species, site, and species × site all *p* < 0.0001). After Bonferroni correction, average body size significantly differs between intraspecific subpopulations of *B. impatiens* and *B. pensylvanicus*. Specifically, for *B. impatiens*, worker body size was larger on average at the urban CC site than at the more rural CW site (contrast of least square means *p* < 0.0001) (Fig. 2). For *B. pensylvanicus* worker body size was larger on average at the rural SNR site than at the more urban CC and ED sites (both contrasts of least square means *p* < 0.0001) (Fig. 2). No other species showed significant spatial structuring of average body size (all contrasts of least square means *p* > 0.006) (Table S2). The Bonferroni adjusted *α*-value used for determining statistical significance between average body size contrasts is *α* = 0.00278 (i.e., 0.05/18 contrasts) (Table S2). We did not include *B. auricomus*, *B. griseocollis*, or *B. pensylvanicus* at CW, and *B. bimaculatus* at CC in these analyses due to ≤ 15 workers having thorax width measurements at these subpopulations. See Table S3 for all worker thorax width sample sizes and body size means per subpopulation.

## Discussion

Studying five bumble bee species across an urban gradient in the greater Saint Louis region, we find evidence for intraspecific spatial structuring of body size, despite genetic homogeneity among subpopulations. Specifically, two species, *B. impatiens* and *B. pensylvanicus*, exhibited body size clines across the urban gradient; however, the direction of these clines were not consistent between species (i.e., sites with increased urbanization were associated with larger *B. impatiens* and smaller *B. pensylvanicus*). As our study sites occur along a gradient from the city of Saint Louis to a rural area west of the city (Fig. 1), these results suggest that human-modified environments can drive body size differences between intraspecific subpopulations of pollinating insects. Notably, we also reaffirm previous reports of low genetic diversity in *B. pensylvanicus*^12,41^ and find evidence that *B. griseocollis*, a species thought to be thriving in North America, may be inbred in the greater Saint Louis region.

Two non-mutually exclusive explanations may account for the observed intraspecific body size clines across the urban gradient: phenotypic plasticity or local adaptation. We argue that our results suggest plasticity underlies the observed phenotypic clines for two primary reasons. First, we did not find evidence for genetic structure in any of our studied species; i.e., all *F*_ST_ values are low (all *F*_ST_ ≤ 0.002; Table 3) and our power analyses indicate that our data had sufficient statistical power to detect true genetic differentiation, if it were present. This suggests high rates of intraspecific gene flow, either currently or in the recent past, throughout the greater Saint Louis region. High rates of gene flow often limit subpopulations from adapting to their local environments, by homogenizing traits throughout a metapopulation^75^. Second, body size is an exceptionally plastic trait in bumble bees, with tenfold differences in body size occurring among highly related intra-colony workers (*r* = 0.75)^15,30^. Plasticity can shield a population from local adaptation by moving the population toward an adaptive peak, thus enabling persistence in a changed environment without adaptive genetic change^76^. Accordingly, the lack of genetic structure, coupled with the known plasticity of bumble bee body size, supports the observed body size spatial structuring being a result of plastic responses to local environments, as opposed to adaptive genetic divergence. However, we cannot definitively rule out the possibility of local adaptation; in rare cases, subpopulations can become locally adapted even while gene flow is maintained^77^. It is possible that recent habitat fragmentation has induced strong differential selection between subpopulations, though sufficient time has not passed for population genetics to reflect this. However, this may be an unlikely explanation of our results, as microsatellites can document genetic effects of recent fragmentation in species of pollinating insects with high gene flow^42^.

Several environmental factors may drive this observed spatial structuring of body size. In bumble bees, worker larvae fed a higher quality diet or at higher rates develop into larger adults^28,29^. It is possible that body size clines in urban environments result from differences in nutritional quality and/or quantity among sites, whereby large size is promoted by high nutritional quality/quantity (or small size results from a constraint of low nutritional quality/quantity). While we did not directly quantify nutrition in this study, our data suggest this may be a likely explanation of our results. First, in all cases where average body size significantly differed between intraspecific sites (i.e., between CC and CW for *B. impatiens* and between SNR and both CC and ED for *B. pensylvanicus*; Fig. 2), conspecific females were observed foraging from a higher richness of floral genera at the sites where body size was larger (Table S4). As bees often optimize nutritional intake by foraging from a variety of floral species^78^, this may correspond to bees having more balanced diets at sites with a higher richness of exploitable floral genera. Second, at all sites where average body size was larger intraspecifically, not only were more floral genera exploited, but colony density was higher as well (Table 1). Numerous studies indicate that colony success is dependent on nutritional availability at a site^78,79^. Thus, the higher colony density observed at sites with a greater richness of exploited floral genera supports the idea that these sites conferred greater nutritional quality and/or quantity. This interpretation—i.e., that the observed body size clines are driven by differences in nutritional quality—would account for the opposite direction of clines found for *B. impatiens* and *B. pensylvanicus*, as long as nutritional quality is greater in urban environments for *B. impatiens*, but is lower in urban environments for *B. pensylvanicus*. Future research should explore the degree of resource partitioning between these two species and whether such partitioning results in contrasting resource quality clines across urban gradients.

It is notable that the greatest magnitude of body size spatial structuring was observed in *B. pensylvanicus*. Numerous reports have suggested *B. pensylvanicus* is the species most at risk of extinction among those studied^43,44^ and our finding of *B. pensylvanicus* having the lowest genetic diversity among bumble bee species throughout the greater Saint Louis region (lowest *AR* in both 2018 and 2017; see Supplemental Materials for description of 2017 population genetics; Table 3; Table S5) reaffirms these reports. Interestingly, however, at SNR—the site where *B. pensylvanicus* was largest intraspecifically and was found feeding from a comparatively high number of floral genera—*B. pensylvanicus* colony density was highest both intraspecifically (i.e., across sites) and interspecifically (i.e., highest interspecific colony density at SNR in both 2018 and 2017) (Table 1 and Table S5). This may suggest that sites with high floral species richness provide robust support to *B. pensylvanicus* populations, thus indicating the potential role that floral enrichment across urbanized landscapes can play in supporting populations of threatened bumble bee species.

The importance of investigating functional trait diversity of threatened species has been increasingly recognized as conservation program efficacy depends on environmental effects on the development and expression of phenotype^33,80^ and plasticity is a primary response of species to global change^4^. The body size clines we observed suggest that human-modified environments can induce landscape-level structuring of developmentally plastic functional traits. Conservation programs should be cognizant of when traits are developmentally, but irreversibly, plastic, as is the case for body size in bumble bees^29,30^. For example, Lema and Nevitt document that pupfish (*Cyprinodon* spp.) exhibit a developmentally plastic small body size as a result of high water temperature and low food availability^35^. They suggest that management programs should consider this by captively breeding pupfish in similar conditions to the population they will be reintroduced to, so that large individuals with high dietary requirements are not reintroduced into a food-limited environment^35^. Similarly, if the body size clines we observed resulted from nutritional differences among sites, this may suggest that spatial structuring of bumble bee body size can be used to indicate variance in environmental quality, with subpopulations with relatively smaller average body sizes being targeted for floral enrichment. However, alternative explanations may underlie the observed body size clines. For example, spatial heterogeneity in environmental contaminants could differentially expose subpopulations to pollutants, which may have downstream effects on foraging behavior^81^ and the development of adult body size^82^. Alternatively, in urban areas, increased metabolic demands imposed by the urban-heat-island (UHI) effect are expected to drive shifts toward smaller body size in certain taxa^83^. The direction of the *B. pensylvanicus* body size cline across the urban gradient follows the predicted direction under the UHI effect, analogous to the Brazilian stingless bee, *Melipona fasciculata*^84^; however, the *B. impatiens* cline follows the opposite pattern. Furthermore, in taxa where body size positively correlates with dispersal distance, habitat fragmentation may drive increased body size to promote movement of individuals between habitat patches^83,85^. Interestingly, Theodorou et al.^86^ found evidence for this trend in *Bombus terrestris* in Central Europe, where workers were larger in cities compared to nearby rural sites, potentially due to urban fragmentation. However, similar to the UHI effect, the contrasting directions of the body size clines found for *B. impatiens* and *B. pensylvanicus* complicate this as a likely explanation for our results. To gain a comprehensive understanding of this system, future studies should directly quantify nutrition, environmental factors, and fragmentation across subpopulations.

Our results exemplify the importance of simultaneously investigating functional trait distributions and conservation genetics of species in urban environments. While *B. griseocollis* does not exhibit spatial structuring of body size throughout the greater Saint Louis area, we find evidence that *B. griseocollis* is potentially inbred in this region. *Bombus griseocollis* had the highest inbreeding coefficient (*F*_IS_) and the second lowest global *AR* among the studied species (Table 3) and 84% of sampled *B. griseocollis* males were diploid in 2018 (Table 2). In haplodiploid bees, males develop via either (1) parthenogenesis, in which hemizygosity at the sex-determining locus produces a viable, haploid male, or (2) a fertilized egg, in which homozygosity at the sex-determining locus produces a sterile, diploid male^71^. As inbreeding promotes an increased proportion of homozygosity^80^, diploid males may occur at higher frequencies in inbred haplodiploid populations. While additional sampling in the Midwest is needed, in replicate years and populations, these results suggest relatively high rates of inbreeding in Saint Louis *B. griseocollis* populations, despite *B. griseocollis* being broadly distributed and abundant throughout much of the United States^87^ and listed as “Least Concern” by the IUCN Red List^43^. Indeed, future research on *B. griseocollis* populations is needed, as understanding why the observed rates of *B. griseocollis* male diploidy are so high will be critical to implementing effective conservation programs. Collectively, our results indicate the utility of simultaneously investigating phenotypic and genetic variation of threatened species, as phenotypic and genetic signatures of population stability can occur independently of one another and together provide a more complete understanding of population stability across heterogeneous landscapes.

## Conclusions

The conservation of threatened species is strengthened by integrative assessments of functional trait variability and population genetics. We document that bumble bees can exhibit intraspecific body size clines in human-modified environments, despite subpopulations being genetically homogenous. These results suggest that urbanization can induce landscape-level structuring of functional traits that are developmentally plastic, potentially due to nutritional differences across sites. We additionally find evidence that (1) *B. pensylvanicus* has comparatively low genetic diversity, reaffirming findings from previous studies^12,41^ and (2) *B. griseocollis* is inbred in the greater Saint Louis region. Collectively, these results are informative for the development of bumble bee conservation programs and add to a growing body of literature on how threatened species are affected by human-modified environments.

## Supplementary Information

Supplementary Information 1. 2017 Female Microsatellite Data.

Supplementary Information 2. 2018 Female Microsatellite Data.

Supplementary Information 3. 2018 Male Microsatellite Data.

Supplementary Information 4. Body Size Data.

Supplementary Information 5. ReadMe File for Supplementary Data.

Supplementary Information 6. Supplemental Material PDF.

## Data Availability

All data used for analyses can be found in the Supplementary Information.
