# Supplementary material for: Bumble bees exhibit body size clines across an urban gradient despite low genetic differentiation
Source: Sci Rep. 2022 Mar 9;12:4166. doi: 10.1038/s41598-022-08093-4 (PMC8907314; doi:10.1038/s41598-022-08093-4)
Supplement: Supplementary file 6 — Supplementary Information 6. Supplemental Material PDF. [file 41598_2022_8093_MOESM6_ESM.pdf]

Scientific Reports

Bumble bees exhibit body size clines across an urban gradient despite low genetic differentiation

Matthew W. Austin[a,b,c,*], Amber D. Tripodi[d], James P. Strange[d,e], and Aimee S. Dunlap[b,c]

[a]Living Earth Collaborative, Washington University, St. Louis, MO , USA
[b]Department of Biology, University of Missouri – St. Louis, St. Louis, MO, USA
[c]Whitney R. Harris World Ecology Center, St. Louis, MO, USA
[d]USDA-ARS-Pollinating Insect Research Unit, Logan, UT, USA
[e]Department of Entomology, The Ohio State University, Columbus, OH, USA

*** Corresponding author:**
Washington University in St. Louis
320 McDonnell Hall, Danforth Campus
St. Louis, MO, USA 63105
AustinMattW@gmail.com
ORCID ID: 0000-0002-1231-9081

Table of Contents

Site Descriptions 2
Power Analysis 3
Amplification Success, Null Alleles, Linkage Disequilibrium, and Hardy-Weinberg Equilibrium 4
2017 Population Genetics: Shaw Nature Reserve 7
Table S1. Microsatellite loci retained for each species 9
Table S2. Contrasts of intraspecific site least-squares means comparisons 10
Table S3. Body size statistics for bumble bee (*Bombus* spp.) workers in the greater Saint Louis region 11
Table S4. Floral genera visited by bumble bee (*Bombus* spp.) females in the greater Saint Louis region in 2018 12
Table S5. Sample sizes, colony estimates, and allelic richness (*AR*) per bumble bee species (*Bombus* spp.) at Shaw Nature Reserve (SNR) in the summer of 2017 13
Fig S1. Global allelic richness at each locus after sample size rarefaction 14
References for Supplemental Material 15

1. Site Descriptions

Calvary Cemetery (CC) is a Catholic cemetery located in the city of Saint Louis, Missouri (MO) (human population density = 1,889 people km^{-2}), which contains 25 acres of prairie managed by the Missouri Department of Conservation along the cemetery's northwestern edge, for which a conservation plan was implemented in 2005 [1]. EarthDance Farms (ED) is an organic farm located in Ferguson, MO (human population density = 1,293 people km^{-2}), comprising 14 acres and a variety of native and agricultural plants, which has been a location of organic food production since 1883 [2]. Castlewood State Park (CW) is a state park adjacent to the Meramac River in Ballwin, MO (human population density = 1,297 people km^{-2}), comprising 1,818 acres of land and was established in 1974 [3]. Shaw Nature Reserve (SNR) is a private nature reserve located on the edge of the Missouri Ozarks in Gray Summit, MO - an unincorporated community near Pacific, MO (human population density = 472 people km^{-2}) - comprising 2,500 acres of land and upwards of eight biomes [4], which was established in 1925 [5].

2. Power Analysis

To ensure that our data had sufficient statistical power to detect true genetic differentiation, we performed a power simulation per species with the program POWSIM 4.1 [6]. POWSIM tests the null hypothesis of no genetic differentiation between subpopulations, given different combinations of sample size, loci, and alleles [6]. Each simulation estimates power via chi-square and Fisher's exact tests, while sampling from populations that diverge following a Wright-Fisher model [6]. For all simulations, we set the expected differentiation between subpopulations to F_{ST}=0.05, which is an appropriate minimum value for true genetic structure [7]. This F_{ST} is equivalent to each subpopulation having N_e=100 after 10 generations of drift [8]. We parameterized each simulation with its respective species' observed sample size, loci number, allele number, and allele frequencies. We ran 1,000 iterations of each simulation with default parameters for dememorizations, batches, and iterations per batch. These simulations indicate the power of our sampling protocol to detect an F_{ST}=0.05 and do not represent the true evolutionary history of our study populations.

3. Amplification Success, Null Alleles, Linkage Disequilibrium, and Hardy-Weinberg Equilibrium

Prior to performing population genetic analyses, we removed loci from our microsatellite data following various quality control measures. Specifically, we removed loci that had ≥20% amplification failure, noisy amplification (making a locus unreliable to score), ≥25% null allele frequency following Chakraborty et al. (1992) [9], or showed significant linkage disequilibrium (LD) with one or more loci. Analyses were performed in R Statistics. The package PopGenReport 2.0 [10] identified null alleles. The package Genepop '007 1.1.4 [11] identified loci in LD. In the following, we describe the results of these quality control measures per species. See Table S1 for loci retained per species for analyses.

3.1 *Bombus auricomus*

We could not reliably score B124, BTern01, and BTMS0062 in *B. auricomus* due to noisy amplification. BT28 exhibited ≥25% null allele frequency. BTern02 showed significant LD with BTMS0052 and BT30 (both $p<0.05$). Accordingly, we removed B124, BTern01, BTMS0062, BT28, and BTern02 from *B. auricomus*. Following these quality measures, 10 loci remained for the population genetic analyses of *B. auricomus.*

3.2 *Bombus bimaculatus*

We could not reliably score BTern02 in *B. bimaculatus* due to noisy amplification. BTMS0083 exhibited ≥25% null allele frequency. The following loci pairs showed significant LD: BTern01 and B96 ($p<0.01$), BT10 and B126 ($p<0.001$), BTMS0062 and BTMS0044 ($p<0.05$), BT28 and BTMS0059 ($p<0.05$). Accordingly, we removed BTern02, BTMS0083, BTern01, BT10,

BTMS0062, and BT28 from *B. bimaculatus*. Following these quality measures, 12 loci remained for the population genetic analyses of *B. bimaculatus.*

3.3 *Bombus griseocollis*

BTern02 and BT30 exhibited ≥20% amplification failure in *B. griseocollis*. We could not reliably score BL15 due to noisy amplification. BTMS0083 showed significant LD with BTMS0066, BTMS0086, and B126 (all $p<0.05$). Furthermore, the following loci pairs all showed significant LD: BTMS0066 and BTMS0062 ($p<0.05$), BTMS0086 and BT28 ($p<0.05$), and BT10 and B96 ($p<0.05$). BTMS0062 showed significant deviation from Hardy-Weinberg equilibrium across populations ($p<0.05$ in the majority of populations). Accordingly, we removed BTern02, BT30, BL15, BTMS0083, BTMS0066, BTMS0086, BT10, and BTMS0062 from *B. griseocollis*. Following these quality measures, 10 loci remained for the population genetic analyses of *B. griseocollis.*

3.4 *Bombus impatiens*

BTern02 exhibited ≥20% amplification failure in *B. impatiens*. We could not reliably score B126 and BTMS0062 due to noisy amplification. BTMS0066 and BTMS0059 exhibited ≥25% null allele frequency. The following loci pairs showed significant LD: B96 and BTern01 ($p<0.05$) and BT30 and BTMS0044 ($p<0.05$). Accordingly, we removed BTern02, B126, BTMS0062, BTMS0066, BTMS0059, B96, and BT30 from *B. impatiens*. Following these quality measures, 11 loci remained for the population genetic analyses of *B. impatiens.*

3.5 *Bombus pensylvanicus*

We could not reliably score BTMS0062, BTern02, and BTMS0044 in *B. pensylvanicus* due to noisy amplification. BTern01 showed significant LD with BL15 and BTMS0081 (both $p<0.05$). Accordingly, we removed BTMS0062, BTern02, BTMS0044, and BTern01 from *B. pensylvanicus*. Following these quality measures, 14 loci remained for the population genetic analyses of *B. pensylvanicus.*

4. 2017 Population Genetics: Shaw Nature Reserve

4.1 *Bumble Bee Sampling*

In addition to the sampling performed in 2018, in the summer of 2017, we sampled worker bumble bees at Shaw Nature Reserve (SNR). From late-June through mid-August, we sampled foraging workers of *B. impatiens*, *B. griseocollis*, *B. auricomus*, and *B. pensylvanicus* by hand-netting 3-4 days per week. After capture, we immediately transferred bees to individual vials containing 100% ethanol. We did not sample *B. bimaculatus* in 2017, as the onset of our sampling corresponded with the latter half of their seasonal period of foraging activity. Sample sizes collected per species can be found in Table S5.

4.2 *Microsatellite Genotyping, Colony Density, and Allelic Richness*

We genotyped all 2017 *Bombus* samples at the USDA-ARS Pollinating Insect - Biology, Management, Systematics Research Unit in Logan, Utah following the same methods as described in the main text for our 2018 samples, with the following exception. For sequencing of these 2017 samples, we transferred 1.2 µL of each PCR product to a new well of a 96 well plate, along with 9 µL of a mixture of 975 µL formamide and 25 µL 500 LIZ (internal size standard). Subsequently, an ABI PRISM 3730 DNA Analyzer at Utah State University's Center for Integrated BioSystems sequenced the samples.

After genotyping our 2017 samples, we performed quality control measures (e.g., removing loci with $\geq$20% amplification failure, noisy amplification, $\geq$25% null allele frequency, significant linkage disequilibrium) and calculated colony density and allelic richness (*AR*) following the methods described in the main text for our 2018 samples. This resulted in a minimum of seven loci

being retained per species for *AR* calculations of 2017 populations. The colony density and *AR* results per species at SNR in 2017 are given in Table S5.

Supplemental Tables

Table S1. Microsatellite loci retained for each species.

Locus	Primer Sequence and Tag	Species				
		Bombus auricomus	*Bombus bimaculatus*	*Bombus griseocollis*	*Bombus impatiens*	*Bombus pensylvanicus*
**B124**[1]	F: *6FAM*-GCAACAGGCGGGTTAGAG<br>R: CAGGATAGGGTAGGTAAGCAG	-	X	X	X	X
**B126**[1]	F: *VIC*-GCTTGCTGGTGAATTGTGC<br>R: CGATTCTCTCGTGTACTCC	-	X	X	-	X
**B96**[2]	F: *PET*-GGGAGAGAAAGACCAAG<br>R: GATCGTAATGACTCGATATG	X	X	X	-	X
**BL11**[3]	F: *PET*-AAGGGTACGAAATGCGCGAG<br>R: TGACGAGTGCGGCCTTTTTC	-	-	-	X	-
**BL13**[3]	F: *PET*-CGAATGTTGGGATTTTCGTG<br>R: GCGAGTACGTGTACGTGTTCTATG	X	X	X	X	X
**BL15**[3]	F: *6FAM*-CGAACGAAAACGAAAAAGAGC<br>R: TCTTCTGCTCCTTTCTCCATTC	X	X	-	-	X
**BT10**[3]	F: *NED*-TCTTGCTATCCACCACCCGC<br>R: GGACAGAAGCATAGACGCACCG	X	-	-	X	X
**BT28**[3]	F: *VIC*-TTGCTGACGTTGCTGTGACTGAGG<br>R: TCCTCTGTGTGTTCTCTTACTTGGC	-	-	X	X	X
**BT30**[3]	F: *PET*-ATCGTATTATTGCCACCAACCG<br>R: CAGCAACAGTCACAACAAACGC	X	X	-	-	X
**BTern01**[3]	F: *VIC*-CGTGTTTAGGGTACTGGTGGTC<br>R: GGAGCAAGAGGGCTAGACAAAAG	-	-	X	X	-
**BTern02**[3]	F: *NED*-TTTCCACCCTTCACGCATACAC<br>R: GATTTTATCCTCCGACCGTTCC	-	-	-	-	-
**BTMS0044**[4]	F: *PET*-AGGATCGAGAGAACGAGCTG<br>R: AGGCCTTGGGAGAGTTCG	X	X	X	X	-
**BTMS0052**[4]	F: *PET*-AAATCCTTCGCTTCCGGTCT<br>R: TGGGGGTAGCAACACTCAAA	X	X	X	X	X
**BTMS0059**[4]	F: *PET*-GGCTAGGAAAGATTAGCACTACC<br>R: AGTTCGACAGACCAAGCTGT	-	X	X	-	X
**BTMS0062**[4]	F: *VIC*-CTGTCGCATTATTCGCGGTT<br>R: CTGGGCGTGATTCGATGAAC	-	-	-	-	-
**BTMS0066**[4]	F: *6FAM*-CATGATGACACCACCCAACG<br>R: TTAACGCCCAATGCCTTTCC	X	X	-	-	X
**BTMS0081**[4]	F: *PET*-ACGCGCGCCTTCTACTATC<br>R: AGGGACACGCGAACAGAC	X	X	X	X	X
**BTMS0083**[4]	F: *6FAM*-CGACTCGTTCGAGCGAAATTA<br>R: GTTTTTGCCAGGCTCCGAAT	-	-	-	X	X
**BTMS0086**[4]	F: *NED*-AGAGAAATTGCATGCGGTCG<br>R: CTCGCGCTTGTCGAATCAAT	X	X	-	X	X

[1]Estoup et al. 1995 [12]; [2]Estoup et al. 1996 [13]; [3]Funk et al. 2006 [14]; [4]Stolle et al. 2009 [15];

X = locus retained; - = locus removed

Table S2. Contrasts of intraspecific site least-squares means comparisons. These contrasts derive from a full analysis of variance (ANOVA) regressing worker body size against bumble bee species (*Bombus* spp.) and site. Statistical significance of contrasts was determined using a Bonferroni corrected α-value (i.e., $p<0.00278$) and is denoted by an asterisk (*) and italicized *p*-value. CC = Calvary Cemetery, CW = Castlewood State Park, ED = EarthDance Farms, SNR = Shaw Nature Reserve.

**Species and Comparison**	**Site Contrast *p*-values**					
	**CC - CW**	**CC - ED**	**CC - SNR**	**CW - ED**	**CW - SNR**	**ED - SNR**
B. auricomus	-	0.6244	0.1279	-	-	0.2808
B. bimaculatus	-	-	-	0.7609	0.0067	0.0215
B. griseocollis	-	0.8305	0.442	-	-	0.3241
B. impatiens	*<.0001**	0.0084	0.0256	0.0114	0.0248	0.9722
B. pensylvanicus	-	0.0105	*<.0001**	-	-	*<.0001**

Table S3. Body size statistics for bumble bee (*Bombus* spp.) workers in the greater Saint Louis region. *N* gives the number of workers included in calculations of body size means. CC = Calvary Cemetery, CW = Castlewood State Park, ED = EarthDance Farms, SNR = Shaw Nature Reserve.

Species and Statistics	Sites			
	CC	CW	ED	SNR
B. auricomus				
N	44	-	34	18
**Mean (95% CI)**	6.40 (6.25-6.55)	-	6.46 (6.34-6.58)	6.60 (6.34-6.86)
B. bimaculatus				
N	-	66	45	28
**Mean (95% CI)**	-	4.34 (4.22-4.46)	4.37 (4.27-4.47)	4.62 (4.49-4.75)
B. griseocollis				
N	41	-	56	23
**Mean (95% CI)**	5.34 (5.23-5.45)	-	5.36 (5.24-5.48)	5.25 (5.00-5.50)
B. impatiens				
N	50	40	63	35
**Mean (95% CI)**	4.83 (4.71-4.95)	4.36 (4.23-4.49)	4.60 (4.48-4.72)	4.60 (4.41-4.79)
B. pensylvanicus				
N	29	-	18	36
**Mean (95% CI)**	5.23 (5.03-5.43)	-	5.59 (5.36-5.82)	6.14 (6.00-6.28)

Table S4. Floral genera visited by bumble bee (*Bombus* spp.) females in the greater Saint Louis region in 2018. The percent of bees visiting each floral genus per species and site are given in parentheses. *n* = number of female bees collected visiting flowers, CC = Calvary Cemetery, CW = Castlewood State Park, ED = EarthDance Farms, SNR = Shaw Nature Reserve.

Species	Floral Genera (Visitation Percent) per Site			
	CC	CW	ED	SNR
	n=49	*n*=0	*n*=39	*n*=43
B. auricomus	*Calystegia* (14.3)<br>*Carduus* (6.1)<br>*Dipsacus* (8.2)<br>*Penstemon* (8.2)<br>*Rumex* (2.0)<br>*Trifolium* (18.4)<br>*Vicia* (42.9)		*Agastache* (5.1)<br>*Monarda* (2.6)<br>*Trifolium* (53.8)<br>*Vicia* (38.5)	*Baptisia* (11.6)<br>*Dasistoma* (4.7)<br>*Iris* (2.3)<br>*Monarda* (58.1)<br>*Penstemon* (20.9)<br>*Pycnanthemum* (2.3)
	n=1	*n*=70	*n*=46	*n*=34
B. bimaculatus	*Ipomoea* (100.0)	*Blephilia* (21.4)<br>*Glechoma* (4.3)<br>*Hydrophyllum* (4.3)<br>*Teucrium* (11.4)<br>*Trifolium* (58.6)	*Agastache* (4.3)<br>*Borago* (2.2)<br>*Lavandula* (2.2)<br>*Salvia* (2.2)<br>*Symphytum* (28.3)<br>*Trifolium* (17.4)<br>*Vicia* (43.5)	*Amorpha* (5.9)<br>*Asclepias* (2.9)<br>*Baptisia* (2.9)<br>*Monarda* (11.8)<br>*Pedicularis* (5.9)<br>*Penstemon* (55.9)<br>*Pycnanthemum* (11.8)<br>*Trifolium* (2.9)
	n=42	*n*=12	*n*=57	*n*=32
B. griseocollis	*Apocynum* (4.8)<br>*Calystegia* (28.6)<br>*Carduus* (4.8)<br>*Dipsacus* (4.8)<br>*Monarda* (2.4)<br>*Securigera* (35.7)<br>*Trifolium* (4.8)<br>*Vernonia* (2.4)<br>*Vicia* (11.9)	*Blephilia* (8.3)<br>*Teucrium* (16.7)<br>*Trifolium* (75.0)	*Agastache* (3.5)<br>*Asclepias* (21.1)<br>*Calystegia* (1.8)<br>*Echinacea* (14.0)<br>*Monarda* (3.5)<br>*Teucrium* (1.8)<br>*Trifolium* (28.1)<br>*Vicia* (26.3)	*Amorpha* (6.3)<br>*Asclepias* (12.5)<br>*Baptisia* (6.3)<br>*Echinacea* (3.1)<br>*Iris* (6.3)<br>*Monarda* (12.5)<br>*Penstemon* (15.6)<br>*Pycnanthemum* (18.8)<br>*Senecio* (3.1)<br>*Veronicastrum* (15.6)
	n=51	*n*=40	*n*=54	*n*=40
B. impatiens	*Calystegia* (23.5)<br>*Cirsium* (31.4)<br>*Dipsacus* (13.7)<br>*Helianthus* (29.4)<br>*Securigera* (2.0)	*Teucrium* (35.0)<br>*Trifolium* (7.5)<br>*Verbesina* (57.5)	*Agastache* (48.1)<br>*Allium* (5.6)<br>*Cichorium* (1.9)<br>*Convolvulus* (1.9)<br>*Ipomoea* (1.9)<br>*Symphyotrichum* (22.2)<br>*Symphytum* (1.9)<br>*Teucrium* (1.9)<br>*Trifolium* (14.8)	*Agastache* (2.5)<br>*Amorpha* (2.5)<br>*Baptisia* (2.5)<br>*Chamaecrista* (7.5)<br>*Dasistoma* (7.5)<br>*Lactuca* (5.0)<br>*Penstemon* (12.5)<br>*Silphium* (2.5)<br>*Solidago* (17.5)<br>*Verbesina* (17.5)<br>*Veronicastrum* (22.5)
	n=38	*n*=5	*n*=17	*n*=53
B. pensylvanicus	*Calystegia* (2.6)<br>*Carduus* (2.6)<br>*Cirsium* (2.6)<br>*Dipsacus* (68.4)<br>*Trifolium* (18.4)<br>*Vicia* (5.3)	*Solanum* (20.0)<br>*Teucrium* (20.0)<br>*Trifolium* (40.0)<br>*Verbesina* (20.0)	*Trifolium* (82.4)<br>*Vicia* (17.6)	*Agastache* (3.8)<br>*Baptisia* (5.7)<br>*Chamaecrista* (5.7)<br>*Coreopsis* (3.8)<br>*Dasistoma* (26.4)<br>*Iris* (3.8)<br>*Lespedeza* (3.8)<br>*Monarda* (5.7)<br>*Penstemon* (5.7)<br>*Scutellaria* (3.8)<br>*Silphium* (26.4)<br>*Vernonia* (1.9)<br>*Veronicastrum* (3.8)

Table S5. Sample sizes, colony estimates, and allelic richness (*AR*) per bumble bee species (*Bombus* spp.) at Shaw Nature Reserve (SNR) in the summer of 2017. N_i is the total number of sampled females, N_g is the number of successfully genotyped females, N_{nr} is the number of colonies detected from genotyping, N_{ns} is the number of colonies standardized for genotyping success, and N_c is colony density. SE = standard error.

**Variable**		**Species**			
		B. auricomus	***B. griseocollis***	***B. impatiens***	***B. pensylvanicus***
**Colony Estimates**	N_i	30	37	47	48
	N_g	29	31	37	46
	N_{nr}	29	31	37	46
	N_{ns}	30.0	37.0	47.0	48.0
	N_c	45.0	55.5	70.5	72.0
***AR* (SE)**		6.40 (1.38)	7.55 (2.03)	7.62 (2.43)	5.56 (2.18)

Supplemental Figure

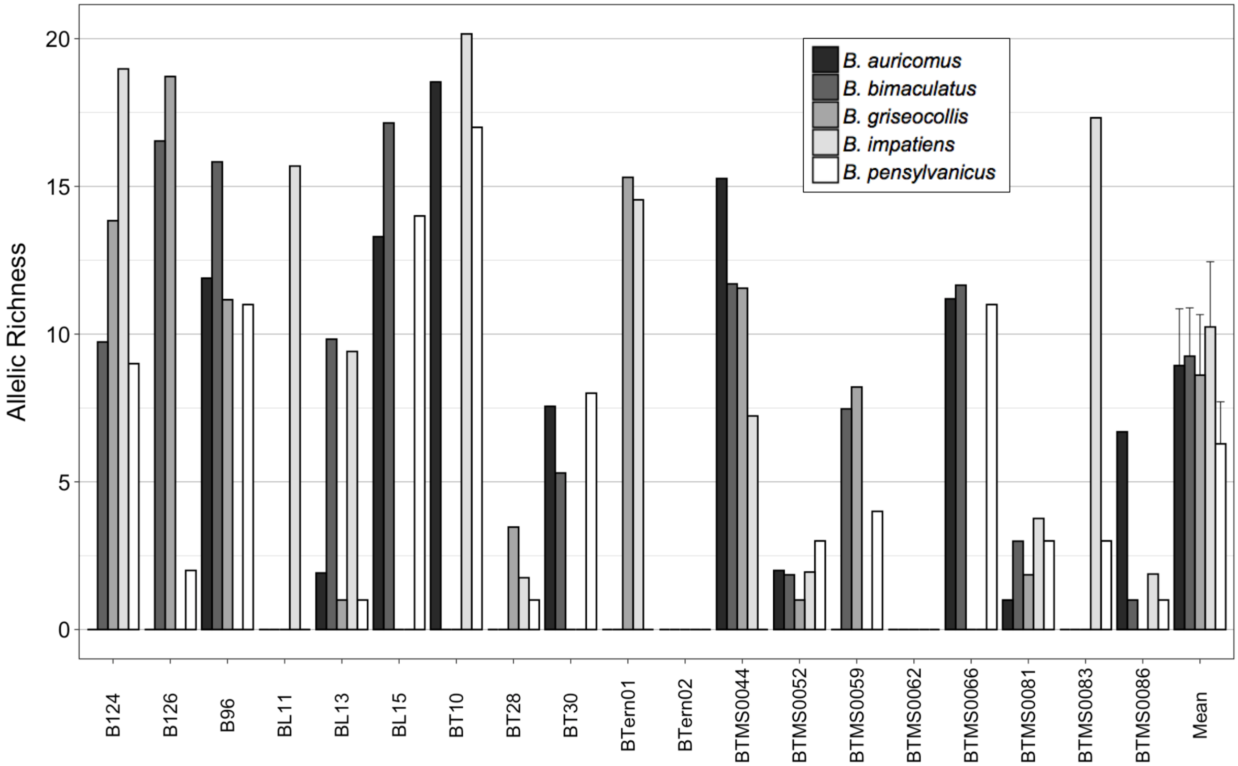

Fig S1. Global allelic richness at each locus after sample size rarefaction (n=176 alleles/species). Loci with zero values were either unamplified or dropped from analyses. Means (+SE) are computed for intraspecific loci with nonzero values.

References for Supplemental Material

1. Bogan, J. Prairie restoration project is in bloom in north St. Louis. St. Louis Post-Dispatch. Accessed 18 Dec. 2019. https://graphics.stltoday.com/apps/prairie/. 2018.

2. EarthDance. About. Accessed 18 Dec. 2019. https://earthdancefarms.org/about/. 2019

3. Wikipedia contributors. Castlewood State Park. Wikipedia, The Free Encyclopedia. Accessed 18 Dec. 2019. https://en.wikipedia.org/w/index.php?title=Castlewood_State_Park&oldid=905158919. 2019a.

4. Missouri Botanical Garden. Plant and Animal Habitats. Accessed 18 Dec. 2019. http://www.missouribotanicalgarden.org/visit/family-of-attractions/shaw-nature-reserve/conservation-at-shaw-nature-reserve/plants-animals-habitats.aspx. 2019.

5. Wikipedia contributors. Shaw Nature Reserve. Wikipedia, The Free Encyclopedia. Accessed 18 Dec. 2019. https://en.wikipedia.org/w/index.php?title=Shaw_Nature_Reserve&oldid=914313203. 2019b.

6. Ryman, N. & Palm, S. POWSIM: a computer program for assessing statistical power when testing for genetic differentiation. *Molecular Ecology Notes* **6**, 600-602 (2006).

7. Frankham, R., Ballou, J.D. & Briscoe, D.A. Introduction to Conservation Genetics. Cambridge University Press, Cambridge. 2002.

8. Nei, M. Molecular Evolutionary Genetics. Columbia University Press, New York, NY. 1987.

9. Chakraborty, R., Andrade, M.D.E., Daiger, S.P. & Budowle, B. Apparent heterozygote deficiencies observed in DNA typing data and their implications in forensic applications. *Annals of Human Genetics* **56**, 45–57 (1992).

10. Gruber, B. & Adamack, A.T. PopGenReport: simplifying basic population genetic analyses in R. *Methods in Ecology and Evolution* **5**, 384-387 (2014).

11. Rousset, F. genepop'007: a complete re-implementation of the genepop software for Windows and Linux. *Molecular Ecology Resources* **8**, 103-106 (2008).

12. Estoup, A., Scholl, A., Pouvreau, A. & Solignac, M. Monoandry and polyandry in bumble bees (Hymenoptera; Bombinae) as evidenced by highly variable microsatellites. *Molecular Ecology* **4**, 89-94 (1995).

13. Estoup, A., Solignac, M., Cornuet, J.M., Goudet, J. & Scholl, A. Genetic differentiation of continental and island populations of *Bombus terrestris* (Hymenoptera: Apidae) in Europe. *Molecular Ecology* **5**, 19-31 (1996).

14. Funk, C.R., Schmid-Hempel, R. & Schmid-Hempel, P. Microsatellite loci for *Bombus* spp. Molecular Ecology Notes **6**, 83-86 (2006).

15. Stolle, E. *et al*. Novel microsatellite DNA loci for *Bombus terrestris* (Linnaeus, 1758). *Molecular Ecology Resources* **9**, 1345-1352 (2009).
